# Supplementary material for: Asking physicians how best to implement cervical cancer prevention services in India: A qualitative study from Mysore
Source: PLOS Glob Public Health. 2022 Jun 2;2(6):e0000570. doi: 10.1371/journal.pgph.0000570 (PMC10021426; doi:10.1371/journal.pgph.0000570)
Supplement: Attachment — Submitted filename: Letterhead_cover letter_PLOSGH_RR.pdf [file pgph.0000570.s001.pdf]

Prajakta Adsul, MBBS, MPH PhD
Assistant Professor, School of Medicine,
University of New Mexico
Address: University of New Mexico
2325 Camino de Salud,
Albuquerque, NM 87131, USA
Phone: 505-272-7351
Email: PAdsul@salud.unm.edu

April 4, 2022

Dear Editors and Reviewers,

We thank you for the opportunity to revise and resubmit our manuscript titled, "Asking physicians how best to implement cervical cancer prevention services in India: a qualitative study from Mysore."

Below you will find our responses (in plain font) to reviewers' comments (in italics). In addressing these comments, we are submitting a revised version for your consideration.

Journal Requirements:

1. *We see that your study includes live participants, but you have not included an Ethics Statement. Please update your manuscript file to include an Ethics Statement subsection to your Materials and Methods section. It should include: i) The full name(s) of the Institutional Review Board(s) or Ethics Committee(s)*

Response: Thank you for your comments, we had this originally in the paper on Page 5, line 122. We have further clarified the sentence.

2. *Please provide separate figure files in .tif or .eps format only. Please ensure that all files are under our size limit of 20MB.*

Response: We have now uploaded the figures in the required format.

Additional Editor Comments (if provided):

3. *Thank you for your submission, and our apologies for the delay in sharing a decision. As you will see from the reviews, there was a split decision between the reviewers between Major and Minor Revisions. Based on R1's comments, we have labelled this as a Major Revision, and invite you to submit a revision responding to Reviewer comments. Thanks again for your patience!*

Response: Thank you!

Reviewer #1:

4. *There is currently limited research in the context of the Indian healthcare system and perceptions of physicians regarding cancer screening services. Most studies to date are quantitative and not qualitative therefore the aim of this study was to use qualitative interviews to understand the perspectives of a group of physicians (PCPs, OBGYN, oncologists, pathologists) involved in cervical cancer related preventative services using the multi-level influences of cancer care continuum model. This is an important and relevant topic. Overall the paper needs re-organization and re-thinking of the emerging themes to interpret the quotations accurately and connect better to the conclusions*.

Response: Thank you for your comments. We hope you will note the extensive revisions to the paper, which we believe have further strengthened the connections to the conclusion and reorganized the paper.

5. *In the methods section, would shift the last few sentences "according to the India's Fourth National....and "to our knowledge" to the Background. A few clarifying questions - are there any OB/GYNs in the public sector in Mysore and if so, why were they excluded? Is it possible to provide a diagram of the snowball sampling to understand from whom the referrals were made (within the public vs. private sectors). Why were pathologists interviewed? They would review samples but not performing any screening. Were the primary care doctors in the public sector specialists that completed residency or medical officers that completed an MBBS (medical school plus intern year)?*

Response: We have moved this sentence to the background. Further, we have clarified that the gynecologists in the study were actually trained OB/GYNs working in the private sector. This is usually the case in the Indian public health sector, there are very few if any trained Ob/Gyns in the public sector. This is the primary reason why the public sector Ob/Gyns did not show up in our snowball sampling approach. As far as the snowball sampling diagram is concerned, we unfortunately did not keep detailed notes

on how the referrals were made and instead focused on keeping records after the participants agreed to participate in the study. This is a point well taken for future studies.

6. *Why were pathologists interviewed? They would review samples but not performing any screening. Were the primary care doctors in the public sector specialists that completed residency or medical officers that completed an MBBS (medical school plus intern year)?*

Response: The screening process includes both delivering the screening tests and the delivery of the test result back to individual patients. For implementing screening programs with success, achieving efficient delivery of both these steps is essential. Based on this rationale, it was important for us to know the process details from the perspectives of the pathologists in the community. In addition, these pathologists came highly recommended by the physicians in terms of their contributions to cervical cancer prevention activities, and per the pathologists, there were several instances when the patients would come directly to a lab to ask for cervical cancer screenings, even without a physician referral. We have now added a clarifying statement, in the methods section about the pathologists. All primary care doctors in the public sector had completed their MBBS (medical school plus intern year).

7. *For the results, I would integrate important quotations into the results and place the larger results table in the Appendix. In general for the quotations, please provide the job title and sector from where the quotations come from.*

Response: We have now removed the table from the manuscript and integrated the quotations into the paper, since there are no word count limits on the manuscripts published in the journal. We have now added the job title and the sector for each supporting quote.

8. *For Theme 5 -- it would be a very concerning major gap if gynecologists reported requiring more training or didn't have a clear sense of when cervical care screenings should be done (as prevention) than pathologists or other physicians.*

Response: We agree with the reviewers. The revisions to theme 5 now clearly indicate the challenges reported by the Ob/gyns in the study, which were related to reporting of Pap smears. We have also added additional quotes from Pathologists corroborating

these data. More specifically, the lack of training was expressed as a gap by the primary care providers, which is also clarified in this section.

9. *A major area of revision for this paper includes: The themes are not always consistent with the quotations and would consider re-wording and re-framing a few themes. It seems like quote 3/quote 4 might have to do with cultural norms, gender inequality and stigma rather than solved by promoting health literacy. Quote 5 seems to describe difficulties with the economic repercussions of trying to access care (missing work). When going into this into the results, would consider splitting the themes and re-organizing since the text under Theme 1 jumps from a lot of different results that are not directly related to Theme 1.*

Response: We have now revised theme 1 to more accurately represent the ideas reflected in the data.

10. *Quote 7 seems to deal with community influence and lack of trust in medical systems more than stigma (possibly more lack of trust in the medical system and community perceptions of what happens at the hospital rather than judgment towards the patient for having cancer). Again, quote 8 seems to be a quote more on not valuing elderly women (not valuing health care services for women and the financial burden of being diagnosed with cancer) rather than stigma towards cervical cancer.*

Response: We have now revised this section to more accurately represent the ideas noted by the reviewer and in the data.

11. *Finally, it's not clear how the multilevel influences on the cancer care continuum was used to organize themes --- would do a better job of tying this framework to the results and conclusions and Figure 1*

Response: For the authors of this paper, Figure 1 visually depicts the embedded nature of healthcare delivery, as outlined in the framework for multilevel influences on the cancer care continuum. We have added this to the discussion section.

Reviewer #2:

12. *The concept is good however some points needs to be explained in more detailed manner. This is with regard to the process of conducting the study. Clarity is to be mentioned regarding the setting, involving opinion of community workers etc. Application and general usability to be implemented also needs to be elaborated.*

THE UNIVERSITY OF NEW MEXICO

Revision of manuscript is needed before consideration for acceptance

Response: In revising the content for the previous reviews, we have substantially strengthened the manuscript in terms of clarity. We have further clarified the methods for the article, in addition to providing additional detail around the discussions. Furthermore, we have revised the results section to appropriately represent the data.

We feel that the review and revision process has resulted in an improved and clarified manuscript. Should you have any questions, feel free to email me at padsul@salud.unm.edu

Sincerely,

Prajakta Adsul, MBBS, MPH, PhD
Division of Epidemiology, Biostatistics, and Preventive Medicine,
Department of Internal Medicine, University of New Mexico Comprehensive Cancer Center

505.272.4180 | Epidemiology, Biostatistics & Preventive Medicine
1 University of New Mexico | MSC10 5550 | Albuquerque, NM 87131
